# Extreme Endurance Migration: What Is the Limit to Non-Stop Flight?

**DOI:** 10.1371/journal.pbio.1000362

**Published:** 2010-05-04

**Authors:** Anders Hedenström

**Affiliations:** Department of Biology, Lund University, Lund, Sweden

Migratory birds have a history of challenging conventional wisdom about the limits of their endurance. More than half a century ago, many ornithologists doubted that a non-stop flight of 860 km across the Gulf of Mexico was possible for migratory (humming) birds [1]. But circumstantial and more direct evidence gathered in the following decade revealed that the Gulf of Mexico is a mere ditch to migratory birds [2],[3], and that some are capable of non-stop flights of up to 5,000 km [4],[5]. And now migratory birds have given their observers reason to pause yet again. In the past year, Gill et al. [6] have provided direct evidence that a shorebird, the Alaskan bar-tailed godwit (*Limosa lapponica baueri*) (Figure 1), makes its eight-day, 11,000-km autumn migration from Alaska to New Zealand in one step, with no stopovers to rest or refuel. This roughly doubles the previous maximum direct flight distance in birds, challenging experts to square this remarkable marathon migration with our understanding of aerodynamic theory and endurance physiology.

**Figure 1:**
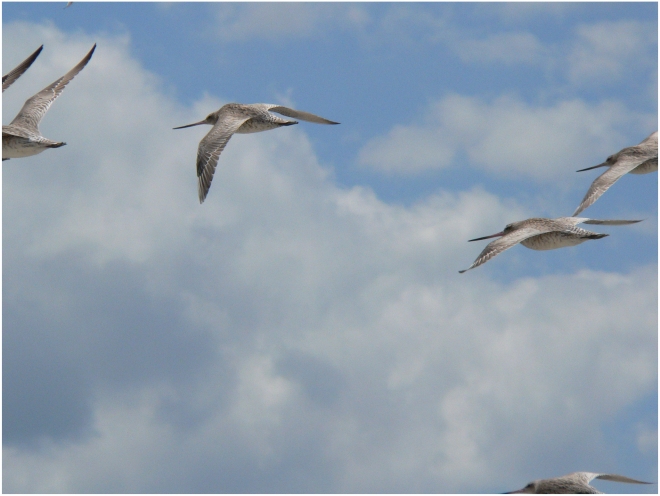
Bar-tailed godwits. Satellite telemetry has recently demonstrated that these birds make one non-stop flight of 11,000 km between Alaska and New Zealand during autumn migration (Image: Phil Battley).

Has this bird finally shattered the limits of long-distance, nonstop migratory flights, forcing researchers to rethink their theories and assumptions about flight and endurance? Or is it possible to show that such feats are possible given what we already understand about aerodynamic theory, metabolism, navigation, and evolution? Here I argue that we already have the tools in hand to understand how it can fly such a distance. What then are the limits to non-stop flight, and can we expect to see these records beaten in the future?

Non-stop flights are common among migratory shorebirds (also known as waders) and often involve trans-oceanic crossings, with examples being American golden plovers (*Pluvialis dominica*) flying 4,000 km between Nova Scotia and South America [7], ruddy turnstones (*Arenaria interpres*) flying 4,000 km between the Pribilof Islands and Hawaii [8], and red knots (*Calidris canutus*) flying 4,800 km between the Wadden Sea and their breeding area on Taymyr [9]. The evidence that the flights are indeed non-stop has often been circumstantial up until now, being based on timing of departure and arrival at main staging sites and how long the journey might take given the amount of fuel accumulated before departure; it is only recently that satellite-based tags have become small enough to allow individual shorebirds to be directly tracked as they migrate [10]. This technique enabled Gill et al. [6] to make their recent observations, and there is more recent circumstantial evidence that sharp-tailed sandpipers (*Calidris acuminata*) make a similar non-stop flight [11] (Figure 2).

**Figure 2:**
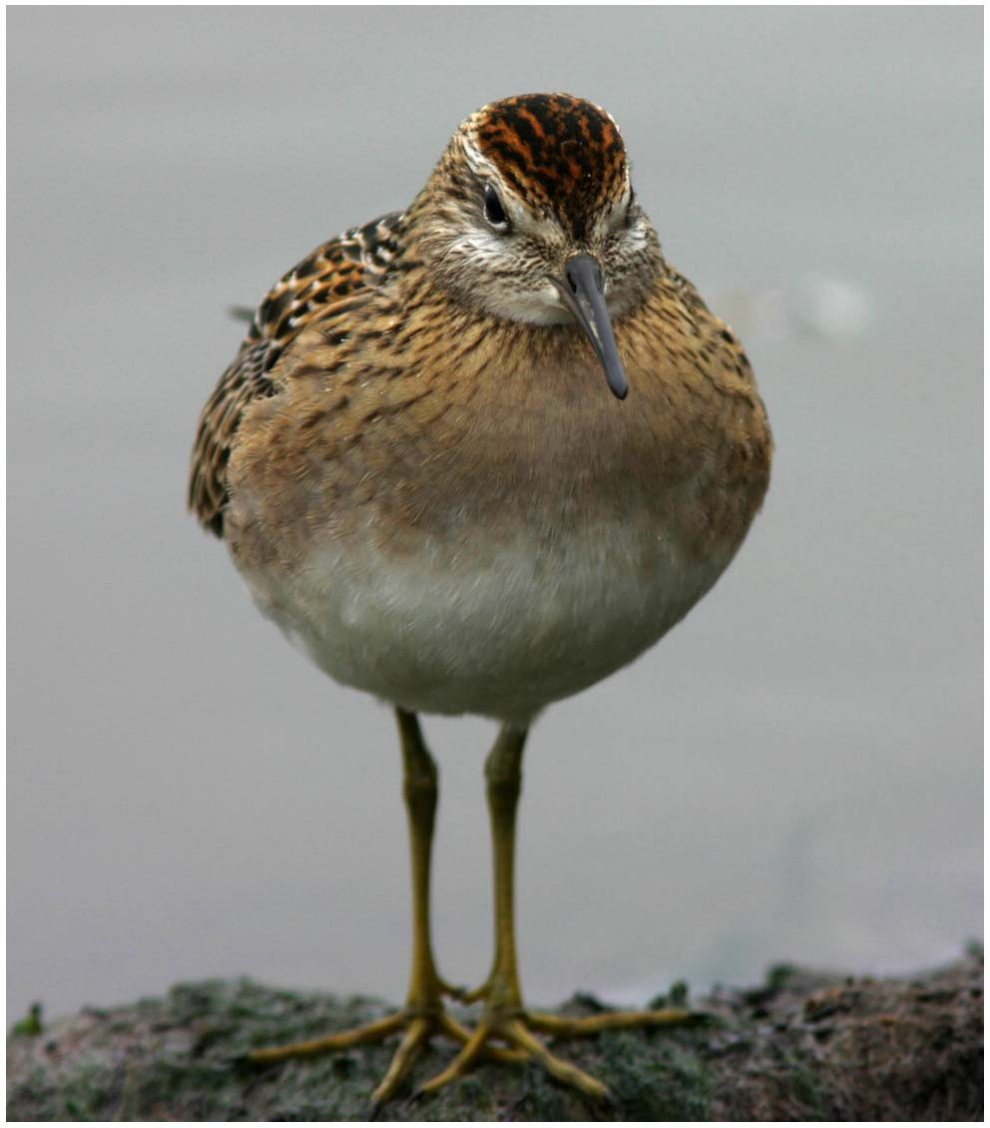
Sharp-tailed sandpiper. Circumstantial evidence suggest that juveniles of this species make a similar non-stop flight as the bar-tailed godwit (Image: Robert E. Gill).

## What Are the Costs of Flight?

To understand how the bar-tailed godwits manage their improbable journey, we first need to know the metabolic costs entailed in long-distance flight. One possibility is that godwits differ in their fuel consumption compared with other birds. The minimum requirement for any long flight (in addition to being able to navigate) is that enough fuel is taken on board before departure to sustain the bird for the duration of the flight; in the godwit's case this is about 8 days, or 192 hours. Godwits weigh 285 g on average without fuel [12]. Assuming that the rate of fuel consumption is a fixed proportion of the current body mass (Box 1), you can show that 0.41% of its body mass is consumed per hour (0.41% m h^−1^, Box 1, Table 1). This is extremely low when compared with previous estimates for passerines and shorebirds more generally, which consume far more of their bodyweight per hour during flight (0.6–1.5% m h^−1^; [13]). However, a similarly low estimate has been made for greater knots (*Calidris tenuirostris*) during their 5,600-km spring migration (0.52% m h^−1^), and for ruddy turnstones during their autumn migration to Hawaii (0.48% m h^−1^, Table 1). Knots and turnstones are a similar size to godwits, which may account for the low proportion of body mass consumption. By comparison, the hummingbird, one of the smallest birds to migrate long distances, has an estimated rate of fuel consumption of 2% m h^−1^, suggesting that they are less efficient than the other larger long-distance migrants.

**Box 1. Flight Range in Flying Vertebrates**

The potential flight range of a bird is primarily given by its departure fuel load, which consists mainly of fat, but also of protein, to some extent. In birds, the fuel is deposited subcutaneously causing an increase in volume (but not length), and thus the projected frontal area increases in proportion to the fuel load, which in turn causes an increased drag from the body. There is also an increased cost of lifting the extra fuel due to gravity. This leads to a diminishing return utility function of added fuel mass because it becomes progressively more expensive to fly with heavy fuel load. To obtain such a function, one can assume that the rate of fuel consumption is a fixed proportion (*x*) of the current body mass (*m*), i.e., *dm/dt*  =  −*xm*, which after integration from starting mass, *m* = (1+*f*)*m*_0_ to the final mass (lean mass *m*_0_) yields the total flying time *T* = (1/*x*)ln(1+*f*). To obtain flight distance the flying time is multiplied by the flight speed *U* to give the range *Y* = *U* (1/*x*)ln(1+*f*). In the case of the Alaskan bar-tailed godwits, we can use the information to estimate *x*, which is a measure of the flight economy. Using *Y* = 11,000 km and *f* = 1.21 (ref. [20]) *x* = 0.0042 (0.42% *m *h^−1^). This is the lowest value obtained thus far for powered animal flight (cf. [13]).

**Table 1:** Estimates of *x* (proportion of body mass used for fuel consumption, see Box 1) for different species of migratory birds and a marine migrant (the eel, *Anguilla anguilla*).

Species	*m*_0_ (kg)	Distance (km)	*X* (% *m* h^−1^)	Source
Blackpoll warbler *Dendroica striata*	0.011	1300	0.56	[36]
Thrush nightingale^a^ *Luscinia luscinia*	0.025	-	1.0	[37]
Bar-tailed godwit *Limosa lapponica*	0.166	11 000	0.42	[6]
Greater knot *Calidris tenuirostris*	0.143	5 400	0.52	[38]
Red knot *Calidis canutus*	0.126	4 800	0.77	[32]
Ruddy turnstone *Arenaria interpres*	0.115	3 700	0.48	[8]
Ruby-throated hummingbird *Archilochus colubris*	0.0044	1 100	2	[3]
Eel *Anguilla anguilla*	0.734	5 500	0.0053	[34]

a based on wind tunnel study.

**Figure 3:**
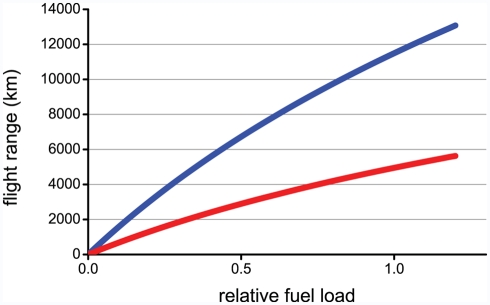
Potential flight range in relation to relative fuel load (expressed as the ratio between fuel mass and lean body mass) for the bar-tailed godwit (blue curve) and the blackpoll warbler (red curve). See Box 1 for details.

Once we know the rate of fuel (mass) consumption, we can then work out the possible duration of the flight, which multiplied with flight speed, gives the distance covered (the flight range). Even if the rate of fuel consumption is quite similar between the blackpoll warbler (*Dendroica striata*) and the godwit, their potential flight range deviates widely because the godwit flies almost twice as fast (Figure 3). According to aerodynamic theory, provided the relative fuel load (i.e., fat and protein stores) is the same, similarly shaped birds should have the same potential flight range irrespective of body size [14]. In real birds, however, this is not the case (Figure 3), because species differ in wing and body shape. For example, the aspect ratio (Box 2) is 5.8 for the blackpoll warbler and 9.3 for the larger godwit, respectively.

**Box 2. Glossary**

**Aspect ratio:** a dimensionless shape index for a wing calculated as the wing span squared divided by wing area (or alternatively the wing span divided by the mean chord, where chord is width of the wing).

**Cost of transport,** ***C*** **:** is calculated as *P*/*mgU*, where *P* is power required during locomotion at speed *U*, *m* is body mass, and *g* is acceleration due to gravity. *C* can be interpreted as the ratio of the average horizontal force needed to push forward to its weight. In aerial locomotion, the inverse of *C* is the well-known lift to drag ratio.

**Great Circle:** a circle that intersects two separate points on a sphere so as to cut it into two equal halves. The shortest arc of the great circle connecting the two points (orthodrome) is the shortest possible path (see Figure 5).

**Isometric:** when changing the size of an object, such as body, keeping the ratio between linear measures, such as length and width, constant is synonymous to isometric or shape preserving scaling.

**Phylogenetic inertia:** the tendency for related species to have similar traits because they both inherited those traits from a common ancestral population.

**Reynolds number (Re):** is a measure of the relative importance of inertial forces to viscous forces. Re is calculated as *Uc*/*ν*, where *U* is speed, *c* is wing chord, and *ν* is kinematic viscosity.

**Rhumb line:** a line between two locations on the earth that crosses all meridians of a longitude at the same angle, i.e., a constant compass course is maintained from the initial bearing (see Figure 5).

**Vortex drag:** a drag that occurs due to the shedding of wing tip vortices by finite wings, which is synonymous to the induced drag due to the induced downwash that lowers the effective angle of attack of the wing.

**Figure 4:**
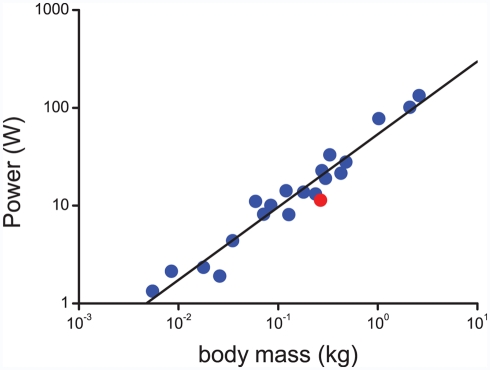
The relationship between flight metabolic power at cruising speed and body mass for a sample of well-studied birds (data compiled in [15]). The bar-tailed godwit is shown as a red symbol estimated from information in [6],[20]. The relationship between power (y-axis) and body mass (x-axis) is y = 53.65x^0.74^, R^2^ = 0.94.

Another factor to consider is the “power consumption” in flight, which is the fuel consumption measured as energy per unit of time (Watts). This energy is provided by metabolizing the fuel substrate, and hence it is related to weight loss. Typically, this is measured as metabolic rate and can be estimated from measures of oxygen consumption either using respirometry masks or by measuring the ratio of oxygen to hydrogen (a “doubly labeled water” technique) in birds flying in wind tunnels. Making the conservative assumption that all mass lost is fat, the godwit has a mid-flight power expenditure of 11.4 W (power is also mass-dependent and will be higher at the beginning and lower at the end of the flight). This estimate, equivalent to running a small light bulb, is shown together with measurements for other bird species in Figure 4. Theoretically, the power required to fly should increase with body mass as 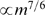
in isometrically scaled (Box 2) birds [14], but in real birds power increases less steeply than this (Figure 4; [15]). Again, this is partly explained by shape differences as we go from small to large birds, but there are also likely to be additional factors that explain the divergence from theory. As Figure 4 indicates, the godwit falls on the low side, but it does not stand out as an extreme outlier compared with other species.

## Fit for Purpose—What Is the Optimal Design for Long-Distance Flight?

Given that bar-tailed godwits have very efficient fuel consumption in general (albeit within a “normal” range), could there be anything else that sets them apart from other birds and thus enable it to fly for eight days non-stop across the Pacific? It is worth noting that this bird's journey far outstrips any manmade flying devices. The current world record for continual flight, held by the solar-powered QiniteQ's Zephyr unmanned aerial vehicle, is 82 hours—half that of the godwits'—and that's with the aid of external solar energy. It does make the godwit's flight seem almost impossible. So how would an engineer design a bird for this purpose?

To begin, the bird must be able to carry a fuel load that lasts for the whole flight. Fuel load-carrying capacity declines with increasing body size [16], which rules out very large birds (say, >1 kg). To minimize lift-induced drag, the wings should be long and slender, which is synonymous with a high aspect ratio (Box 2). The godwit has an aspect ratio of 9.3, which is medium-high, but by no means exceptional among shorebirds.

Another important feature for extreme endurance is a well-streamlined body shape, which helps to reduce the drag created by the body. In the godwit study, transmitters were surgically implanted in females, thus the beneficial effect of the body's streamlining was not severely disrupted. Two males who were fitted with externally attached transmitters, however, failed to reach New Zealand, presumably because of elevated drag. Independent of shape, drag also declines with increasing Reynolds number (see Box 2), because increasing micro scale turbulence in the boundary layer prevents or postpones flow separation over the body with the effect of reducing the chaotic (and costly) wake. The bar-tailed godwit operates, therefore, within a critical region where drag often drops drastically because of a sudden transition to turbulence in the boundary layer (at a Reynolds number of order 10^5^) [17].

Flight speed per se is another important factor for a well-designed flying machine (Box 1), provided that fuel consumption is kept low. Apart from arriving at its destination more quickly, a high flight speed is advantageous because it makes the bird relatively immune to crosswinds, which will cause it to drift off-course. Theoretically, characteristic flight speed increases with body size (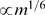
_,_ [14]), and so according to this criterion, the optimal design is a large bird. Indeed, shorebirds, which are relatively large, do have comparatively high flight speeds among birds [18].

In addition to these purely engineering considerations, a bird can increase its flight efficiency further by getting rid of its digestive organs (by atrophy) before long flights to reduce redundant bodyweight not needed for the journey (“non-fuel payload mass,” [19],[20]). Indeed, during long flights, even flight muscles are partially consumed. The reason for this is not fully understood: either muscle protein is used for maintenance or repair of other organs [21] or it is fine-tuned to declining power requirements, so that the whole bird gets successively lighter during the flight. In another long-distance flying shorebird, the semipalmated sandpiper (*Calidris pusilla*), the amphipod food eaten just before departure appears to contain fatty acids that may increase the aerobic capacity of the birds, which almost seems to be comparable to self-doping [22]. Finally, shorebirds fly in flock formation, which potentially could save some energy [23],[24], but for a bird using flapping flight, as the godwit does, rather than gliding, such gains, if any, are bound to be small.

Clearly, there are several factors that contribute to the high flight efficiency and outstanding flight range in bar-tailed godwits, the combination of which result in the bar-tailed godwit being quite close to the “optimal design.” But many of these factors are present in other shorebirds as well and none stand out in the godwit as being exceptional on their own. Even if this species is bending the limits of performance, I think such aerodynamics can still be reconciled within current models of flight performance and physiology.

## Keeping to the Right Path?

We know that the godwit displays no exceptional design features, but it still flies twice the distance of many other migrants. Perhaps the clue to the godwits success, therefore, is in its ability to navigate across an ocean during a week of non-stop flying?

Research on avian senses has shown that direction cues can be obtained from stellar objects (stars, the moon, the sun and its related skylight polarization pattern) combined with a time-keeping mechanism, the earth's magnetic field or olfactory cues (e.g., [25]). It is also known from experiments that information from one type of compass can be calibrated for another. For example, in nocturnally migrating thrushes (*Catharus ustulatus*), information from the sun at twilight is transferred to the magnetic compass on a daily basis [26]. However, it is still largely unknown how birds use orientation cues while flying, especially during flights as long as the godwits'. Do they follow some travel-plan involving directional shifts or do they try to fly in a constant heading (a fixed compass bearing) from the site of departure by using local cues?

**Figure 5:**
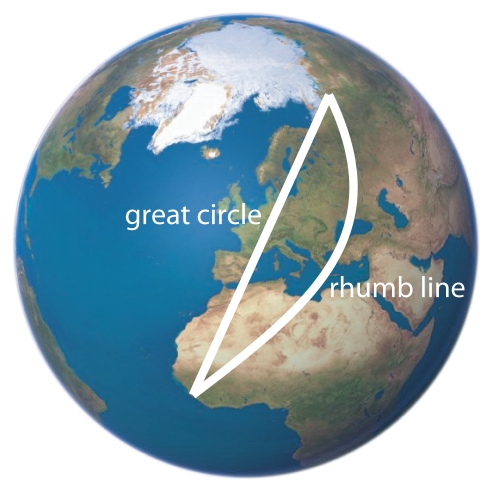
Illustration of how the great circle (orthodrome) and rhumb line (loxodrome) differ between two locations on the spherical globe. The rhumb line is a constant compass course from the point of departure to the destination, while the great circle involves a continuous shift of the compass direction. On the global view shown here, great circles appear as straight lines if passing through the centre.

At high latitudes, a constant compass direction (without re-setting their internal clock to local time) would allow birds to fly along “great circles” (Box 2, Figure 5) if the migration direction has an East–West component [27]. This involves the bird making continuous shifts of direction, but has the advantage of resulting in the shortest route (Box 2, Figure 5). Such shifts are possible at high latitudes using the sun's azimuth as direction cue, where distances between longitudes are small. Here, a shift in course of approximately 1° for each degree of longitudinal displacement matches the change in the sun's azimuth associated with the difference in local time between longitudes. Hence, the birds can exploit the fact that they become “jet lagged” to their advantage. However, the godwits fly along a largely North-South axis, which is within the same time zone, and here the “rhumb line” (Box 2) and the great circle are very similar. The expected difference in departure directions between the rhumb line and great circle between Alaska and New Zealand is so small (189° versus 195°), that the observed mean of 193° could refer to either, leaving no clue as to the direction to follow.

Would it, therefore, be sufficient to maintain a constant heading throughout 7–8 days of flying and rely on the assumption that variable winds will cancel out during such a long journey? Like many other birds, the godwits depart with tail winds, but these will only assist the birds during the initial phase of their journey. Around the equator, the birds will encounter the trade winds that go in opposite directions north and south of the equator (and which therefore roughly cancel out if you cross both). Simulations have shown that birds flying between eastern North America and northeastern South America across the West Atlantic may do so by keeping a constant heading and thus let predictable winds carry them to their specific destination [7]. Godwits fly to different islands between Australia and New Zealand, so the target is quite a large area. Because individual godwits show very diverse flight tracks, such wind-directed flights seem a likely strategy for them.

How the birds go about maintaining their orientation, however, remains a mystery. If the magnetic compass is an inclination compass (i.e., measures the dip angle between the magnetic field lines and the horizontal), it will not provide information at the equator where the field lines are horizontal. However, some birds seem capable of picking up useful information in a nearly vertical magnetic field deviating less than 2° from vertical [28], and so they are perhaps limited only during a relatively short distance around the equator when birds must rely on alternative (celestial) cues.

It seems clear that figuring out the mechanisms birds use to navigate and orient during such marathon migrations—for example, what cues they use to maintain orientation during long-distance flights, how often they check the compass(es), and how and if they integrate available information—will require moving beyond the traditional laboratory-based paradigm of experimentally manipulating one cue at a time. We need novel approaches such as tracking wild birds on the wing combined with experimental manipulation (e.g., in-flight manipulation of visual or magnetic cues). The current satellite telemetry tracks of the godwits are not yet of fine enough resolution to determine small shifts of direction in their course, which could indicate a new compass reading. GPS-based telemetry gives improved route accuracy [29], which may be used to evaluate orientation mechanisms and behavioral responses to local conditions.

## How Did Such Long-Distance Migration Evolve?

**Figure 6:**
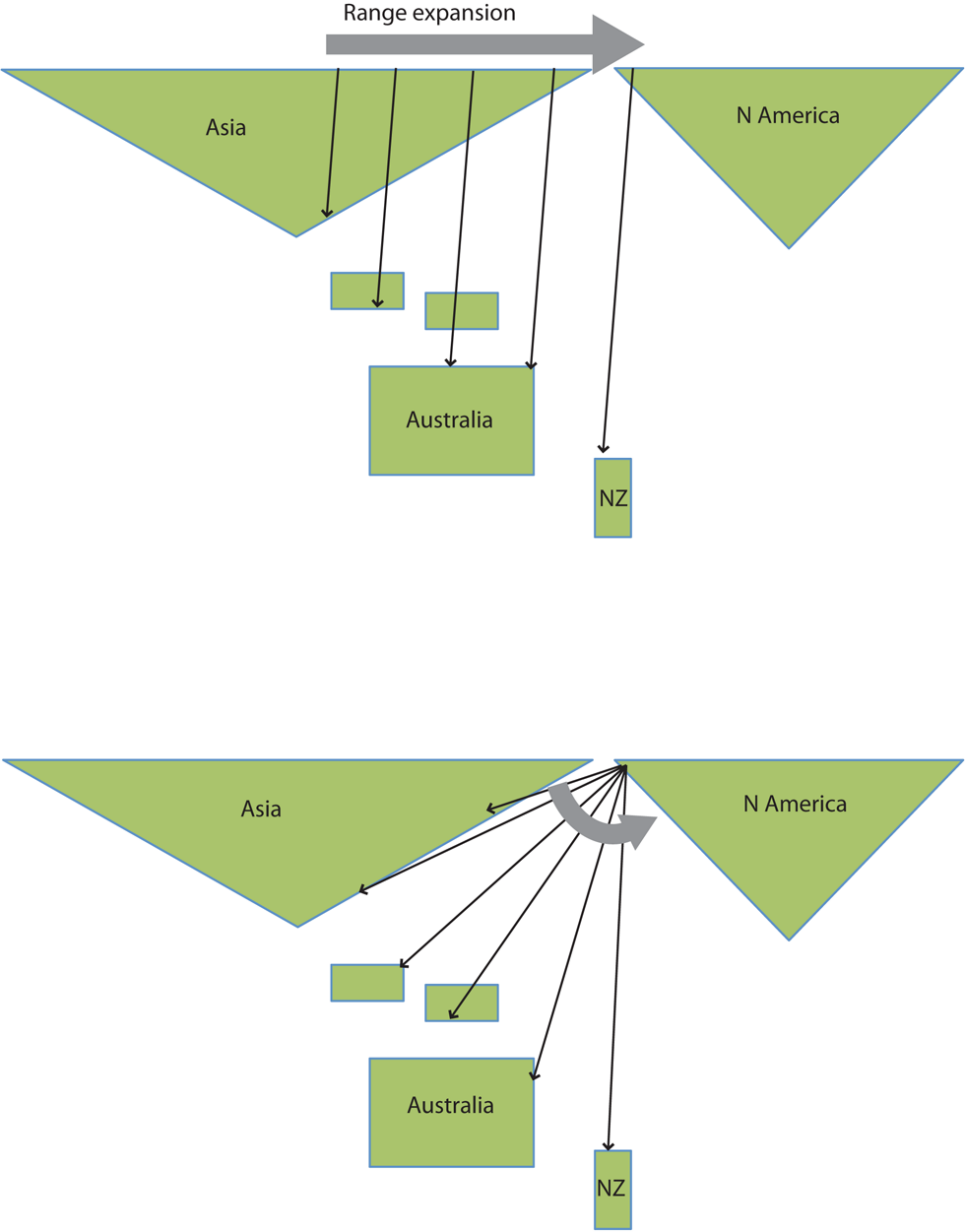
Two hypothetical scenarios about the evolution of long-distance migration in bar-tailed godwits breeding in Alaska. (Top) A breeding range expansion with maintained migration direction but increased distances. (Bottom) An increased migration direction paired with shifted migration distance.

Why long-distance migration evolves in the first place is a complex question [30], and the trans-oceanic flights of Alaskan bar-tailed godwits represent one extreme end of the spectrum. It is unlikely that naïve short distance migratory birds accidentally reached New Zealand to establish this migration route, since that would have required excessive “incidental” fat deposits to keep them going for the duration of the flight. Hudsonian godwits (*Limosa haemastica*) do however visit New Zealand occasionally, but these are already long-distance migrants (albeit less than the bar-tailed godwit) with wintering areas in southern South America and have probably accompanied flocks of bar-tailed godwits in Alaska [12]. As with other seemingly improbable adaptations, it is most likely that the Alaska–New Zealand autumn migration route evolved gradually (Figure 6). One hypothetical scenario is that there was already a long-distance migrating population breeding in Central Siberia and wintering in South Asia. The population expanded towards the east while prolonging its migration, initially via wintering sites on the Philippines, the Indonesian islands and/or New Guinea, Australia and eventually reaching New Zealand (Figure 6A). A second scenario assumes an Alaskan breeding population with a short-range migration to wintering sites in Northeast Asia gradually extending to South Asia. As long-distance migration between Alaska and South Asia became more common, a continuous shift of the migrants to wintering sites further to the east and south eventually established a direct flight route to New Zealand (Figure 6B). Mapping the historical distribution of bar-tailed godwits with molecular genetic information could perhaps resolve whichever of these, or other scenarios, is the most likely candidate. During spring, the godwits split their migration in at least two stages via a detour to East Asia, having a staging post in the Yellow Sea area, before flying to the breeding areas in Alaska. This looped migration argues in favor of scenario two (Figure 6B) and for the evolution of a direct migration route in the autumn.

Evolution of long-distance migration in birds appears, therefore, a rather plastic behavior that can change relatively quickly and evolve independently in different taxa (i.e., with little phylogenetic inertia, [31], Box 2). This is probably because many birds are pre-adapted for migration [32], i.e., they already possess the sensory and physiological capacities needed.

## Are There No Limits to Long-Distance Migration?

The bar-tailed godwit is clearly a highly efficient migrant among birds, probably bending the physiological limits to the extreme. However, locomotion by swimming is even more cost-efficient than flying [33]. The eel (*Anguilla anguilla*) migrates between Europe and the Sargasso Sea, a distance of 5,500 km. The energy consumption during a simulated version of this migration can be measured in a flow tank [34]. The rate of body mass loss due to swimming was as low as 0.005% h^−1^ (Table 1), which is 80 times lower than in the godwit. The dimensionless cost of transport for the eel and the godwit is 0.04 and 0.4, respectively, i.e., it is 10 times cheaper to move a unit weight eel over a unit distance than it is to move a godwit. But there is one big disadvantage to being an eel—travel speed. It would take the eel 690 days to cover the godwit roundtrip migration.

Can we expect the bar-tailed godwit record of a 11,000-km non-stop flight to be broken? I would guess not, simply because the physical limitations of the Earth do not offer any combination of ecologically feasible breeding and wintering areas more distantly apart that would require longer flights. There are potentially longer migrations than that of the Alaskan bar-tailed godwit, such as that of the pectoral sandpiper (*Calidris melanotos*) breeding in Central Siberia and wintering in South America (a distance of 16,000 km), but this migration is broken up into at least two flights. Arctic terns (*Sterna paradisaea*) also perform an impressive 24,000-km northbound spring migration from Antarctica to Greenland during 40 days [35], but these sea birds have the opportunity to feed at sea as they go. Hence, it seems likely that the Alaskan bar-tailed godwit will keep its position as the number one non-stop long-distance flyer.

Even if it has now been confirmed that the bar-tailed godwits do perform mindboggling direct flights across the Pacific, and that we do not need to rethink our theories and assumptions about flight and endurance to explain it, these flights raise many new questions. How do birds orientate while flying? Do they respond and compensate for crosswinds? What factors determine the flight altitude? Answering these and many other mysteries of extreme long-distance migrations will require new innovative techniques to track migrating animals, and, importantly, to record and/or manipulate conditions as they go. Moreover, as fascinating as the question to scientists may be of how these migrating birds acquired the suite of adaptations necessary to perform their marathon migrations, the issue is not strictly academic. Engineers, who have so far managed to develop only aeronautic shadows of the boundary-busting feats of natural fliers, have much to learn from avian innovations most wonderful if they hope to design airborne crafts in their image.
